# Bone and Skin/Subcutaneous Tissue Concentrations of Cefiderocol During Treatment of Extensively Drug-Resistant *Pseudomonas aeruginosa*

**DOI:** 10.1093/jbcr/irae026

**Published:** 2024-02-29

**Authors:** Scott W Mueller, Kyle C Molina, Brittany Blass, Cameron Gibson, Amber D Kohler, Martin Krsak, Arek J Wiktor

**Affiliations:** Department of Pharmacy, University of Colorado Hospital, Aurora, CO 80045, USA; Department of Emergency Medicine, University of Colorado Anschutz School of Medicine, Aurora, CO 80045, USA; Division of GI, Trauma, and Endocrine Surgery (GITES), Department of Surgery, University of Colorado Hospital, Aurora, CO 80045, USA; Division of GI, Trauma, and Endocrine Surgery (GITES), Department of Surgery, University of Colorado Hospital, Aurora, CO 80045, USA; Division of GI, Trauma, and Endocrine Surgery (GITES), Department of Surgery, University of Colorado Hospital, Aurora, CO 80045, USA; Division of Infectious Diseases, Department of Medicine, University of Colorado School of Medicine, Aurora, CO 80045, USA; Division of GI, Trauma, and Endocrine Surgery (GITES), Department of Surgery, University of Colorado Hospital, Aurora, CO 80045, USA

**Keywords:** cefiderocol, concentration, penetration, bone, skin/subcutaneous tissue

## Abstract

Pyoderma gangrenosum is a rare dermatologic disorder that disrupts the skin barrier, requiring immunosuppressive therapy. We successfully used cefiderocol for the treatment of an extensively drug-resistant *Pseudomonas aeruginosa* bacteremia, and presumed osteomyelitis in a patient with severe pyoderma gangrenosum and associated immunosuppressive therapy while being medically optimized for skin grafting. We obtained bone and skin/subcutaneous tissue while the patient was on cefiderocol under an institutional review board-approved biologic waste recovery protocol. Cefiderocol concentrations in bone and skin/subcutaneous tissue were 13.9 and 35.9 mcg/g, respectively. The patient recovered from bacteremia and underwent autografting without further complications. Cefiderocol at approved dosing of 2 g IV (3-hour infusion) every 8 hours resulted in bone and skin/subcutaneous tissue concentrations adequate to treat extensively drug-resistant Gram-negative bacteria that remain susceptible to cefiderocol.

## INTRODUCTION

Pyoderma gangrenosum (PG) is a complex neutrophilic dermatosis with multiple syndromic subtypes that can cause a spectrum of severity. As an inflammatory disorder, the cornerstone of PG treatment includes immunosuppression, a risk factor for infection.^1,2^ Cefiderocol (Fetroja, Shionogi Inc. Florham Park, NJ) is an innovative cephalosporin with siderophore properties, forming complexes with ferric iron that enable it to traverse the outer cell membrane and carry out its bactericidal action by inhibiting cell wall synthesis.^3^ This pharmacological characteristic endows cefiderocol with effectiveness against a wide variety of clinically significant multidrug-resistant Gram-negative bacteria, including carbapenem-resistant *Pseudomonas aeruginosa*.^4^ Cefiderocol was approved by the United States Food and Drug Administration in 2019 for the treatment of complicated urinary tract infections and subsequently approved for the treatment of hospital-acquired bacterial pneumonia and ventilator-associated bacterial pneumonia.^5^ It is approved by the European Medicine Agency for the treatment of infections caused by Gram-negative bacteria in adult patients with limited treatment options.^6^ Limited data have described the concentrations of cefiderocol in several clinically important tissues not evaluated in previous studies including bone. We report cefiderocol concentrations in bone and skin/subcutaneous tissue recovered from a patient undergoing a dermal autograft procedure with severe PG and presumed osteomyelitis.

## METHODS

This research was conducted under a secondary use protocol (Colorado Multiple Institution Review Board 22-0113), allowing biological waste specimens to be collected and analyzed with waived patient consent. Although not required, the patient verbally consented and was in agreement with sample collection and analysis procedures throughout his course of care. Plasma samples were collected on 4 consecutive days that met collection criteria (immediately frozen following clinical laboratory analysis). Tissue and bone chips from the scalp generated as medical waste were collected intra-operatively during a debridement performed as part of routine medical care. All samples were stored at −80 °C. All samples were analyzed by Keystone Bioanalytical, Inc. (North Wales, PA www.keystonebioanalytical.com) via LC/MS/MS.

## CASE

In brief, a 48-year-old male with a complex medical history suffering from severe PG developed left frontal sinus osteomyelitis and mastoiditis shown on CT imaging. He was initiated on ampicillin-sulbactam and doxycycline. Immunosuppression for his PG was initiated with infliximab 10 mg/kg and planned at approximately 3-week intervals. During this time, the patient became colonized on skin wounds with extensively drug-resistant (XDR) *P. aeruginosa,* susceptible only to amikacin (prior to CLSI M 100-Ed33), tobramycin, and cefiderocol (Table 1). After the third dose of infliximab, high-dose methylprednisolone was required for eye swelling. Five days following steroid exposure, the patient became febrile. Blood cultures were obtained, and cefiderocol 2-grams IV (3-hour infusion) every 8 hours was initiated. Creatinine clearance by Cockcroft–Gault equation ranged from 98 to 65 mL/min (confirmed 80 mL/min by a direct urine collection measurement). XDR *P. aeruginosa* bacteremia was confirmed (3/4 cultures, 2 strains Table 1). The patient remained hemodynamically stable throughout the treatment course and was maintained on infliximab to suppress pathergy in preparation for surgical autografting of the head and scalp 15 days after initiating cefiderocol. By request, plasma was frozen by the clinical lab after applicable tests were performed. Four of the 7 requested biologic waste samples were deemed to be appropriate for drug concentration analysis, unfortunately, the day of surgery sample was not immediately frozen and therefore not available for analysis. Although minimal biologic waste resulted from surgery, scalp bone fragments, and skin/subcutaneous tissue were recovered. The resulting cefiderocol concentrations are presented in Table 2. The patient remained on cefiderocol for 4 weeks (10 days following autografting). The patient was discharged following the successful surgery on postoperative day 12.

**Table 1.** Antimicrobial Susceptibility Testing of *Pseudomonas aeruginosa* Blood Cultures

	*Pseudomonas aeruginosa* 1*	*Pseudomonas aeruginosa* 2
Amikacin**	S	S
Aztreonam	R	I
Cefepime	R	R
Ceftazidime	R	S
Ceftolozane/Tazobactam	I	S
Ciprofloxacin	I	I
Imipenem	R	R
Levofloxacin	R	R
Meropenem	R	R
Piperacillin/Tazobactam	R	S
Tobramycin	S	S
Ceftazidime/Avibactam MIC (mcg/mL)***	R 64	
Cefiderocol MIC (mcg/mL)****	S 0.5	S 0.5

S, susceptible; I, intermediate; R, resistant; MIC, minimum inhibitory concentration; mcg, microgram; mL, milliliter.

Testing performed by Kirby–Bauer disk diffusion unless otherwise specified.

* Carba-R PCR negative; multiple isolates recovered with identical susceptibility pattern, stable cefiderocol MIC.

** Susceptibility prior to CLSI M 100-Ed33.

*** E-test.

**** Broth microdilution at Laboratory Specialists, Inc. (Westlake, OH) and ARUP Laboratories (Salt Lake City, Utah).

**Table 2.** Cefiderocol Concentrations in Bone, Skin/Subcutaneous Tissue and Plasma

Sample source	Day of therapy	Time from end of infusion (min)	Cefiderocol concentration
Bone	18	248	13.9 mcg/g
Subcutaneous tissue/skin	18	218	35.9 mcg/g
Plasma 1	21	329	26.5 mcg/mL
Plasma 2	22	225	43.7 mcg/mL
Plasma 3	23	275	45.9 mcg/mL
Plasma 4	24	227	47.8 mcg/mL

min, minutes; mcg, microgram; mL, milliliter.

## DISCUSSION

Among other interesting aspects of this case to be described elsewhere, we demonstrated the successful treatment of XDR *P. aeruginosa* bacteremia with cefiderocol in an immunosuppressed patient without reduction of his therapeutic immunosuppression for PG, as the underlying disease pathology involved skin barrier disruption. The patient remained stable throughout his bacteremia largely due to an early selection of an effective antimicrobial. Given the severity of his skin disorder, surveillance skin cultures were collected which allowed us to identify the most appropriate antibiotic agent for a multi-drug-resistant pathogen such as XDR *P. aeruginosa* in advance of an invasive infection.

Our case is novel in the fact that we have analyzed plasma, bone, and soft tissue concentrations of cefiderocol. Although we do not have plasma drug concentrations at the time of bone and tissue collection to infer direct plasma-to-tissue distribution, the amount of cefiderocol in bone and tissue suggests adequate penetration to the compartments of interest at 248 and 218 minutes after the end of a 3-hour infusion when at steady state. Furthermore, the observed plasma concentrations we report appear similar to mean plasma concentrations reported near these time points in other PK studies which suggests a substantial degree of penetration to bone and skin/subcutaneous tissue is achieved with typical doses.^7,8^ Given the increasingly common threat of multi-drug-resistant Gram-negative bacteria, especially in the immunosuppressed and burn population, reporting antibiotic concentrations in difficult-to-penetrate compartments remain important to guide appropriate antimicrobial selection and dosing. Our case demonstrates that cefiderocol adequately penetrated bone at 2-g IV every 8 hours (3-hour infusion) to treat multi-drug-resistant Gram-negative bacteria otherwise susceptible to cefiderocol.

This case is not without limitations. Ideally, we would have cefiderocol plasma concentrations at the time of tissue collection. This prevents a definitive interpretation of plasma to bone or tissue penetration. Our secondary use protocol only allows for the recovery of biological waste generated by clinically indicated procedures. Data generated through this process limits sample collection to procedures needed for patient care only and to the availability of samples that would otherwise be discarded. Nevertheless, these samples represent a steady state (>14 days of therapy), and one may reasonably interpret the results as presented. It is possible that cefiderocol degradation occurred in blood samples between collection, clinical laboratory assessment, and freezing. We do not believe this process negatively impacted the interpretation as plasma concentrations over multiple days were both consistent and within expected values. Lastly, the tissue and bone samples were not suspected to be directly infected. We cannot comment on the amount of cefiderocol in infected bone, though the acutely infected bone is believed to have a higher degree of blood perfusion and thus potential medication penetration.^9,10^ Regardless, we believe these estimates to be clinically useful.

## CONCLUSION

Early active antimicrobial therapy with cefiderocol effectively treated XDR *P. aeruginosa* bacteremia in an immunosuppressed patient with severe pyoderma gangrenosum. Bone and tissue cefiderocol concentrations suggest adequate drug penetration to the respective compartments at FDA-labeled doses to treat multi-drug-resistant Gram-negative bacteria otherwise susceptible to cefiderocol.

## Notes

*Author contributions:* Scott W. Mueller (Conceptualization [lead], Data curation [lead], Funding acquisition [equal], Investigation [lead], Methodology [lead], Project administration [lead], Resources [lead], Writing—original draft [lead], Writing—review & editing [equal]), Kyle C. Molina (Data curation [equal], Formal analysis [equal], Writing—original draft [equal], Writing—review & editing [equal]), Brittany Blass (Investigation [equal], Writing—review & editing [equal]), Cameron Gibson (Methodology [supporting], Writing—original draft [supporting], Writing—review & editing [supporting]), Amber D. Kohler (Data curation [supporting], Writing—review & editing [supporting]), Martin Krsak (Data curation [supporting], Investigation [supporting], Writing—original draft [supporting], Writing—review & editing [supporting]), and Arek J. Wiktor (Conceptualization [equal], Data curation [equal], Funding acquisition [equal], Investigation [equal], Project administration [equal], Writing—original draft [equal], Writing—review & editing [equal])

*Funding:* This work was supported by an investigator-initiated grant by Shionogi, Inc. [grant number 22-0113-230683] (PI: A.J.K. and S.W.M.; <10,000 USD). A.J.K.’s institution received related indirect fees.
